# Supplementary material for: Mixed-Lineage Leukemia 1 Inhibition Enhances the Differentiation Potential of Bovine Embryonic Stem Cells by Increasing H3K4 Mono-Methylation at Active Promoters
Source: Int J Mol Sci. 2023 Jul 25;24(15):11901. doi: 10.3390/ijms241511901 (PMC10418322; doi:10.3390/ijms241511901)
Supplement: Supplementary file 1 [file ijms-24-11901-s001.zip › ijms-2476711-supplementary.pdf]

Mixed-Lineage Leukemia 1 Inhibition Enhances the Differentiation Potential of Bovine Embryonic Stem Cells by Increasing H3K4 Mono-Methylation at Active Promoters

Chen Li[1], Xuejie Han[1], Jing Wang[1], Fang Liu[1], Yuanyuan Zhang[1], Zihong Li[1], Zhenyu Lu[1], Yongli Yue[1], Jinzhu Xiang[1], and Xueling Li[1*]

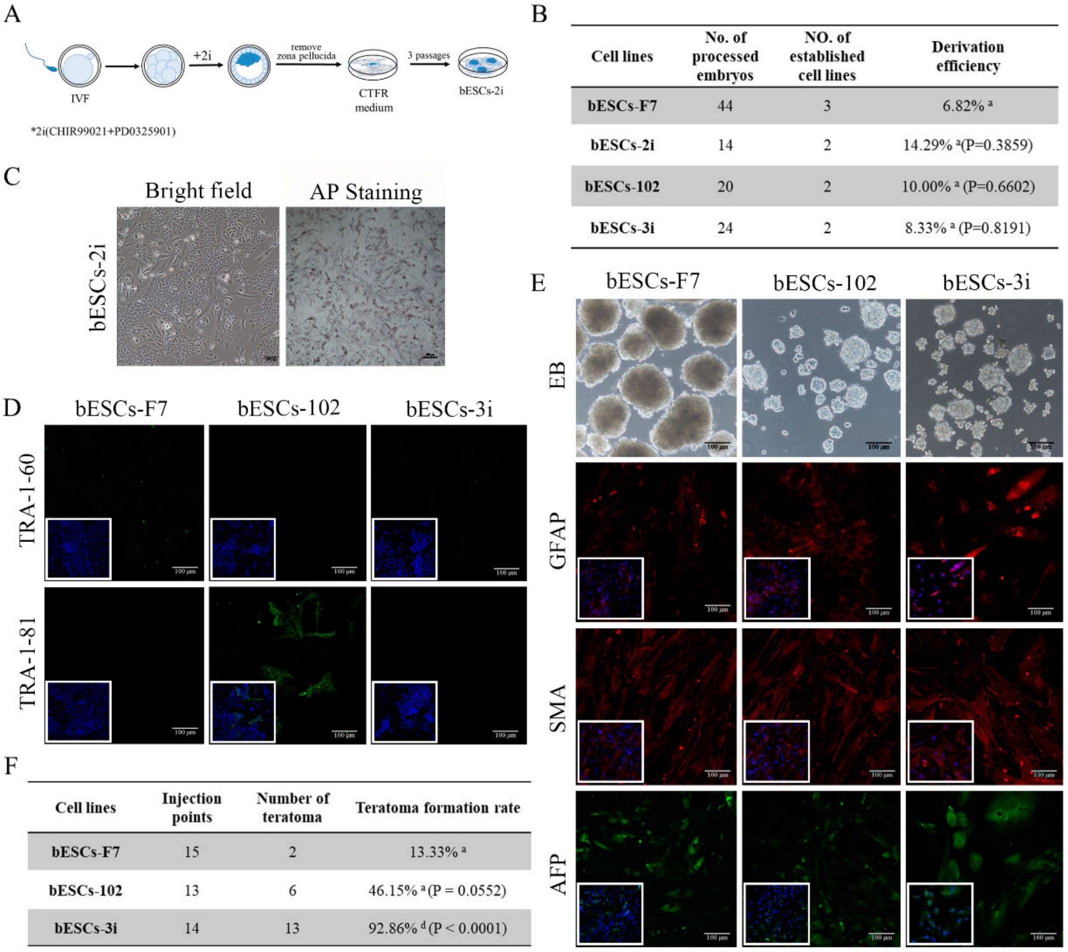

Cell lines	No. of processed embryos	NO. of established cell lines	Derivation efficiency
bESCs-F7	44	3	6.82% a
bESCs-2i	14	2	14.29% a(P=0.3859)
bESCs-102	20	2	10.00% a (P=0.6602)
bESCs-3i	24	2	8.33% a (P=0.8191)

Cell lines	Injection points	Number of teratoma	Teratoma formation rate
bESCs-F7	15	2	13.33% a
bESCs-102	13	6	46.15% a (P = 0.0552)
bESCs-3i	14	13	92.86% d (P < 0.0001)

Figure S1. Establishment of bESCs from bovine blastocysts treated with different combination of inhibitors and comparison of pluripotency and differentiation ability of derived bESCs.

(A) Schematic diagram of established bESCs − 2i. (B) Comparison of bESCs generation rates of bESCs − F7, bESCs − 2i, bESCs − 102 and bESCs − 3i. P − values of the blastocyst rate were determined by the chi − squared test with Yates' correction, with the bESCs − F7 group as control. Values in the same column with the same letters (a, a) indicate no significant difference (P > 0.05). (C) The morphology and alkaline phosphatase staining of bESCs − 2i (scale bar, 200 μm). (D) IF of pluripotency transcription factors TRA − 1 − 60 and TRA − 1 − 81 of bESCs − F7, bESCs − 102 and bESCs − 3i (scale bar, 100 μm). (E) bESCs − F7, bESCs − 102 and bESCs − 3i spontaneously differentiate into EBs *in vitro* (Scale bar, 100 μm). IF staining is performed after differentiation. GFAP (ectoderm), SMA (mesoderm) and AFP (endoderm) (scale bar, 200 μm). (F) Comparison of teratomas formation rates of bESCs − F7, bESCs − 102 and bESCs − 3i. P values of the blastocyst rate were determined by the chi − squared test with Yates' correction, with the bESCs − F7 group as control. Values in the same column with the same letters (a, a) indicate no significant difference (P > 0.05).

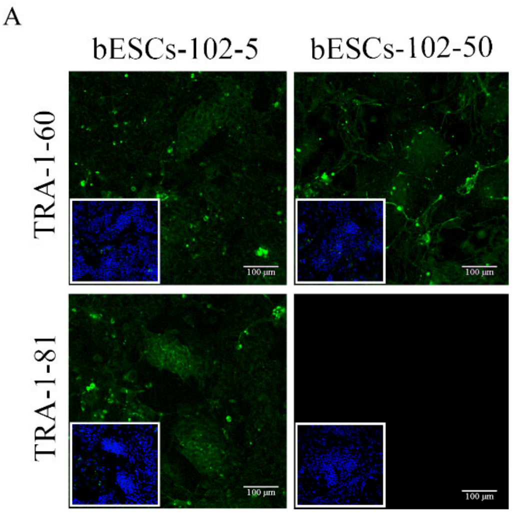

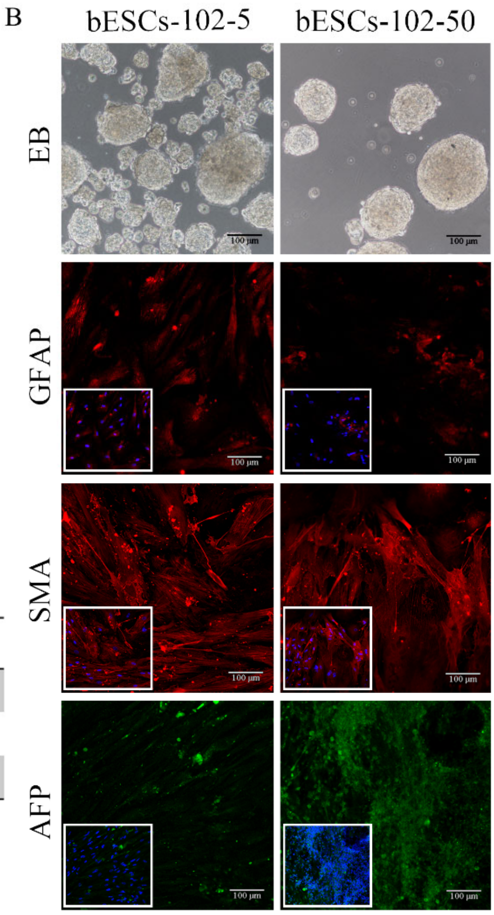

C

Cell lines	Injection points	Number of teratoma	Teratoma formation rate
bESC-F7	15	2	13.33% [a]
bESCs-102-5	19	5	26.32% [a] (P = 0.3526)
bESCs-102-50	17	5	29.41% [a] (P = 0.2722)

Figure S2. The pluripotency and differentiation ability of bESCs after MLL1 inhibition.

(A) IF of pluripotency transcription factors TRA − 1 − 60 and TRA − 1 − 81 of bESCs − 102 − 5 and bESCs − 102 − 50 (scale bar, 100 μm). (B) bESCs − F7, bESCs − 102 − 5 and bESCs − 102 − 50 spontaneously differentiate into EBs in vitro (scale bar, 100 μm). IF staining is performed after differentiation. GFAP (ectoderm), SMA (mesoderm) and AFP (endoderm) (scale bar, 200 μm). (C) Comparison of teratomas formation rates of bESCs − F7, bESCs − 102 and bESCs − 3i. P values of the blastocyst rate were determined by the chi − squared test with Yates' correction, with the bESCs − F7 group

as control. Values in the same column with same letters (a, a) indicate not significantly difference (P > 0.05).

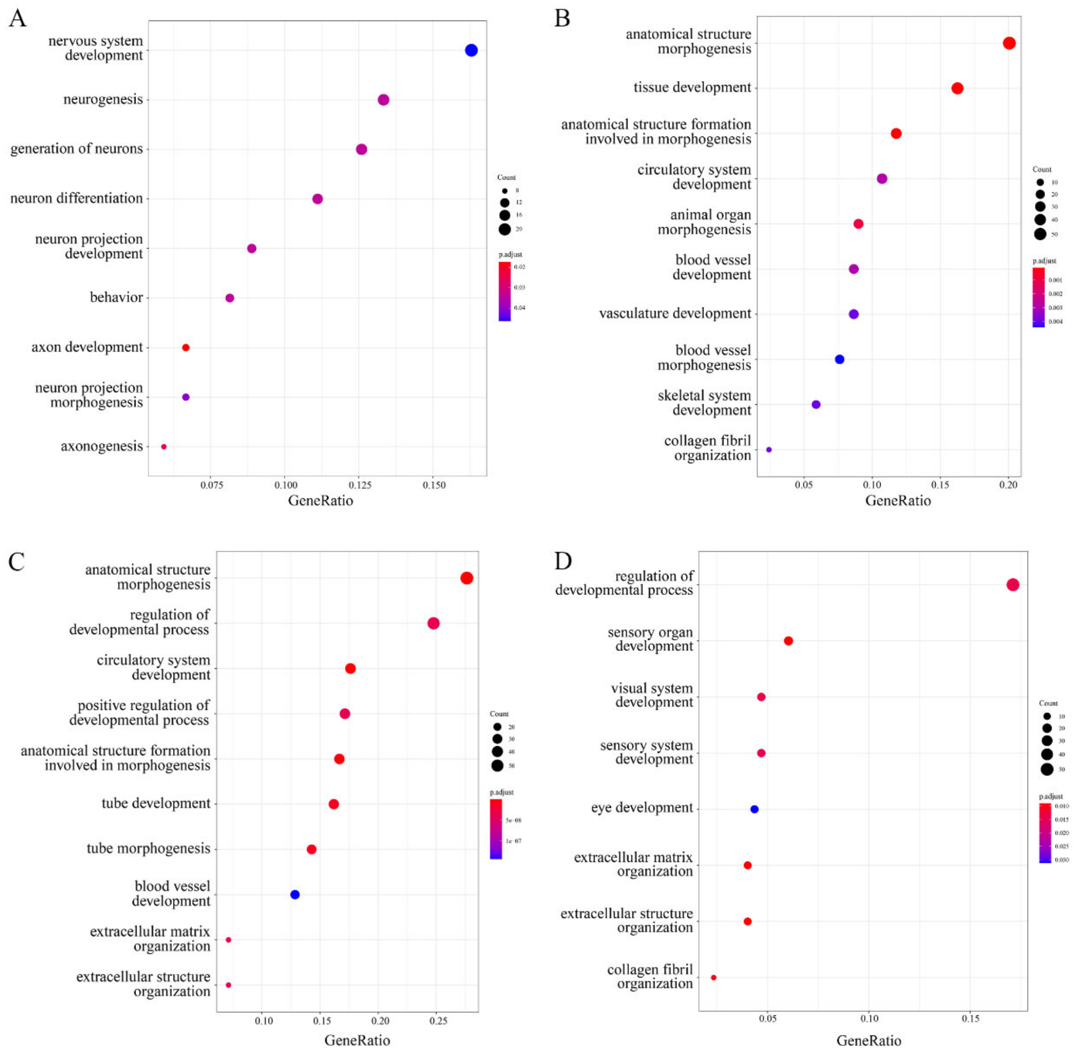

Figure S3. GO biological process analyses of bESCs

(A) GO biological process terms of differentially genes between bESCs − F7 and bESCs − 102. Up − regulated genes in bESCs − 102. (B) GO biological process terms of differentially genes between bESCs − F7 and bESCs − 102. Down − regulated genes in bESCs − 102. (C) GO biological process terms of differentially genes between bESCs − F7 and bESCs − 3i. Down − regulated genes in bESCs − 3i. (D) GO biological process terms of differentially genes between bESCs − F7 and bESCs − 102 − 5. Down

－regulated genes in bESCs－102－5.

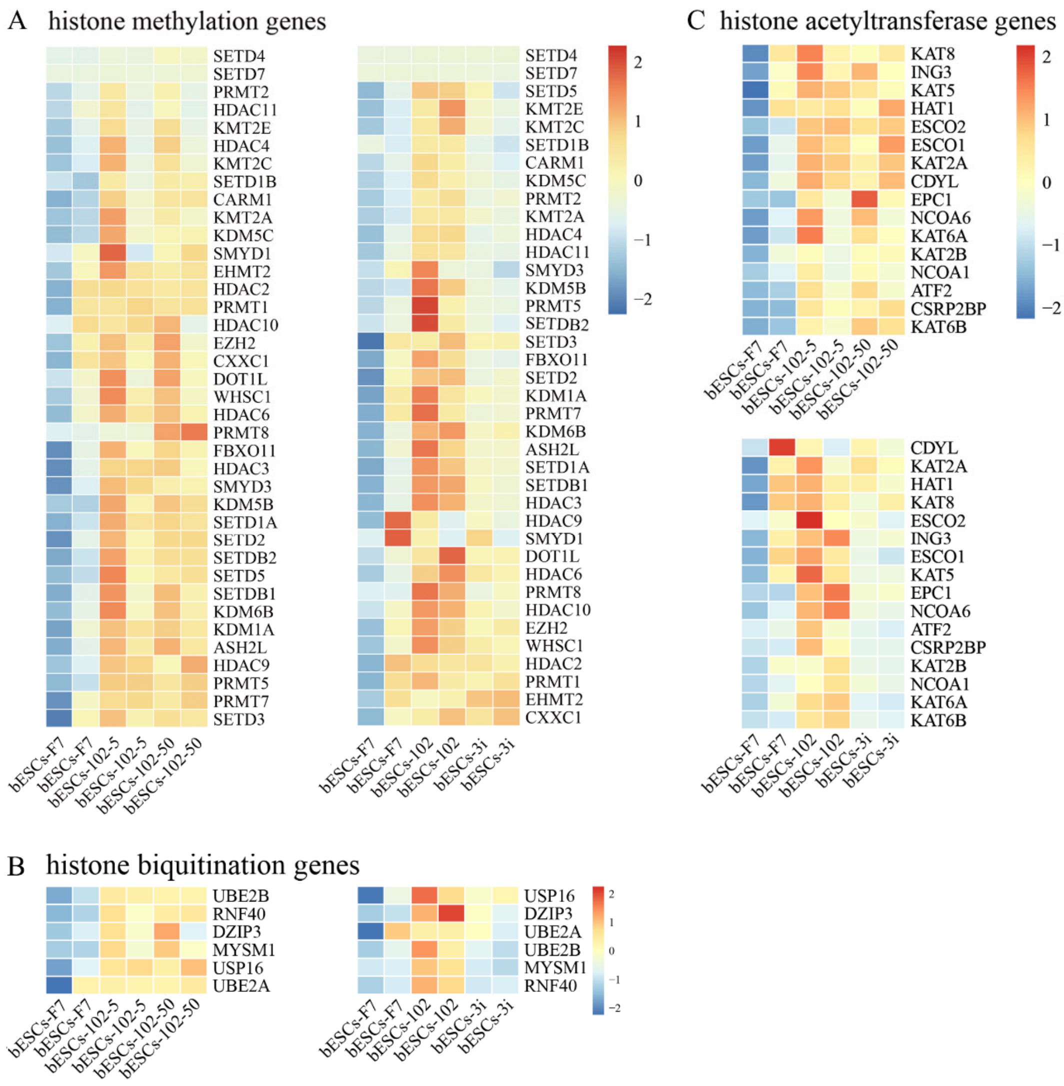

Figure S4. MLL1 inhibition up－regulates the expression of regulatory genes associated with epigenetic modification.

(A) Heat map of histone methylation genes in bESCs. RNA－seq was performed. RPKM values were used to define up－regulated genes (RPKM≥1, red) and down－regulated genes (RPKM <1, blue). (B) Heat map of histone ubiquitination genes in bESCs. RNA－seq was performed. RPKM values were used to define up－regulated genes (RPKM≥1, red) and down－regulated genes (RPKM <1, blue). (C) Heat map of

histone acetyltransferase genes in bESCs. RNA − seq was performed. RPKM values were used to define up − regulated genes (RPKM≥1, red) and down − regulated genes (RPKM <1, blue).

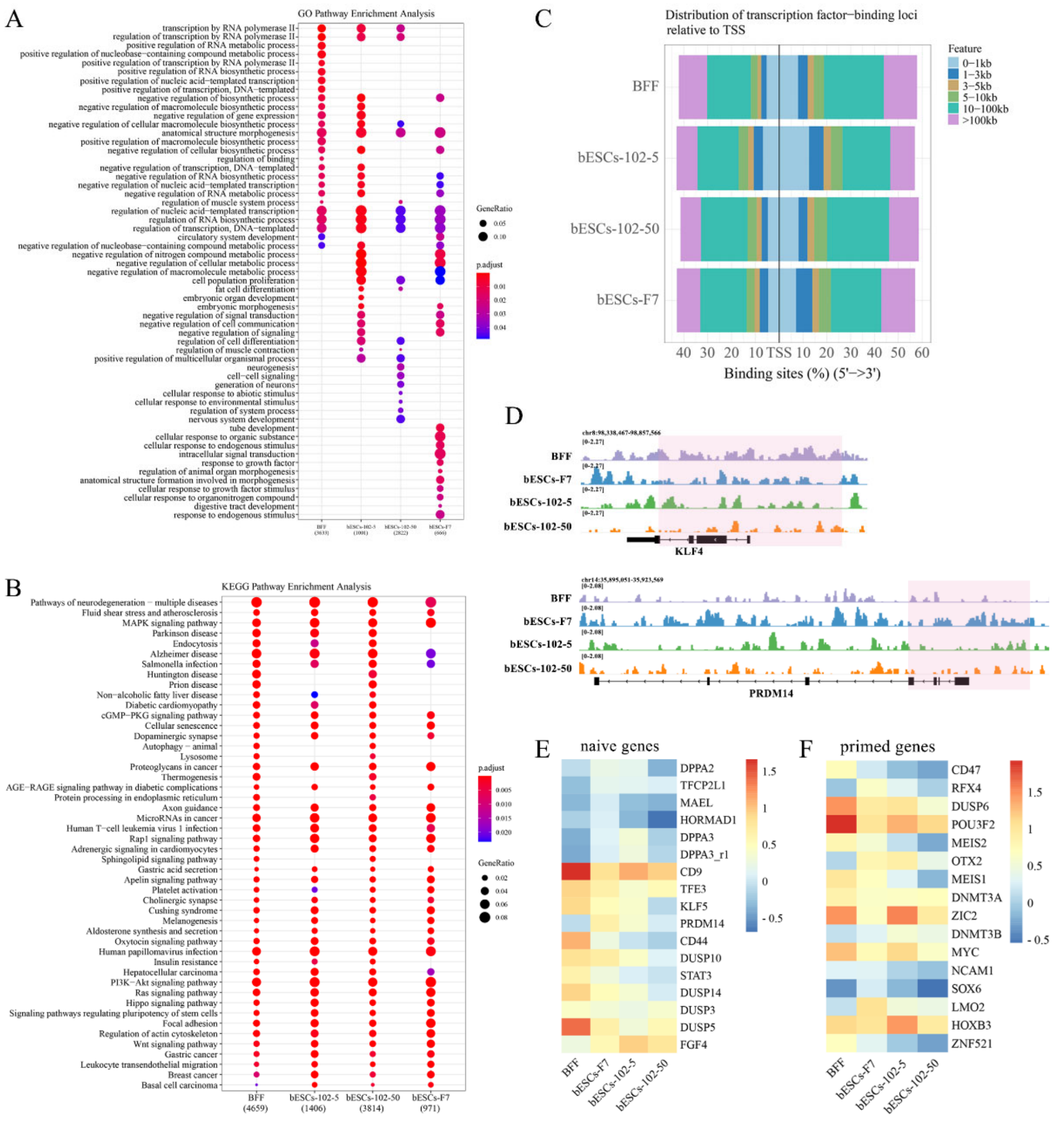

Figure S5. MLL1 inhibition changes the H3K4me1 distribution of bESCs − F7.

(A) GO biological process terms of H3K4me1 ChIP − seq of bESCs − F7, bESCs − 102 − 5, bESCs − 102 − 50 and BFF. (B) KEGG of H3K4me1 ChIP − seq of bESCs − F7,

bESCs − 102 − 5, bESCs − 102 − 50 and BFF. (C) Ratios of bESCs − F7, bESCs − 102 − 5, bESCs − 102 − 50 and BFF gene regions based on H3K4me1 ChIP − seq. (D) Peak map of H3K4me1 enrichment in genes of KLF4 and PRDM14 of bESCs − F7, bESCs − 102 − 5 and bESCs − 102 − 50 promoter sites (pink regions) comparison. (E) H3K4me1 ChIP − seq analysis of naïve pluripotency markers. RPKM values were used to define up − regulated genes (RPKM≥1, red) and down − regulated genes (RPKM <1, blue). (F) H3K4me1 ChIP − seq analysis of primed pluripotency markers. RPKM values were used to define up − regulated genes (RPKM≥1, red) and down − regulated genes (RPKM <1, blue).

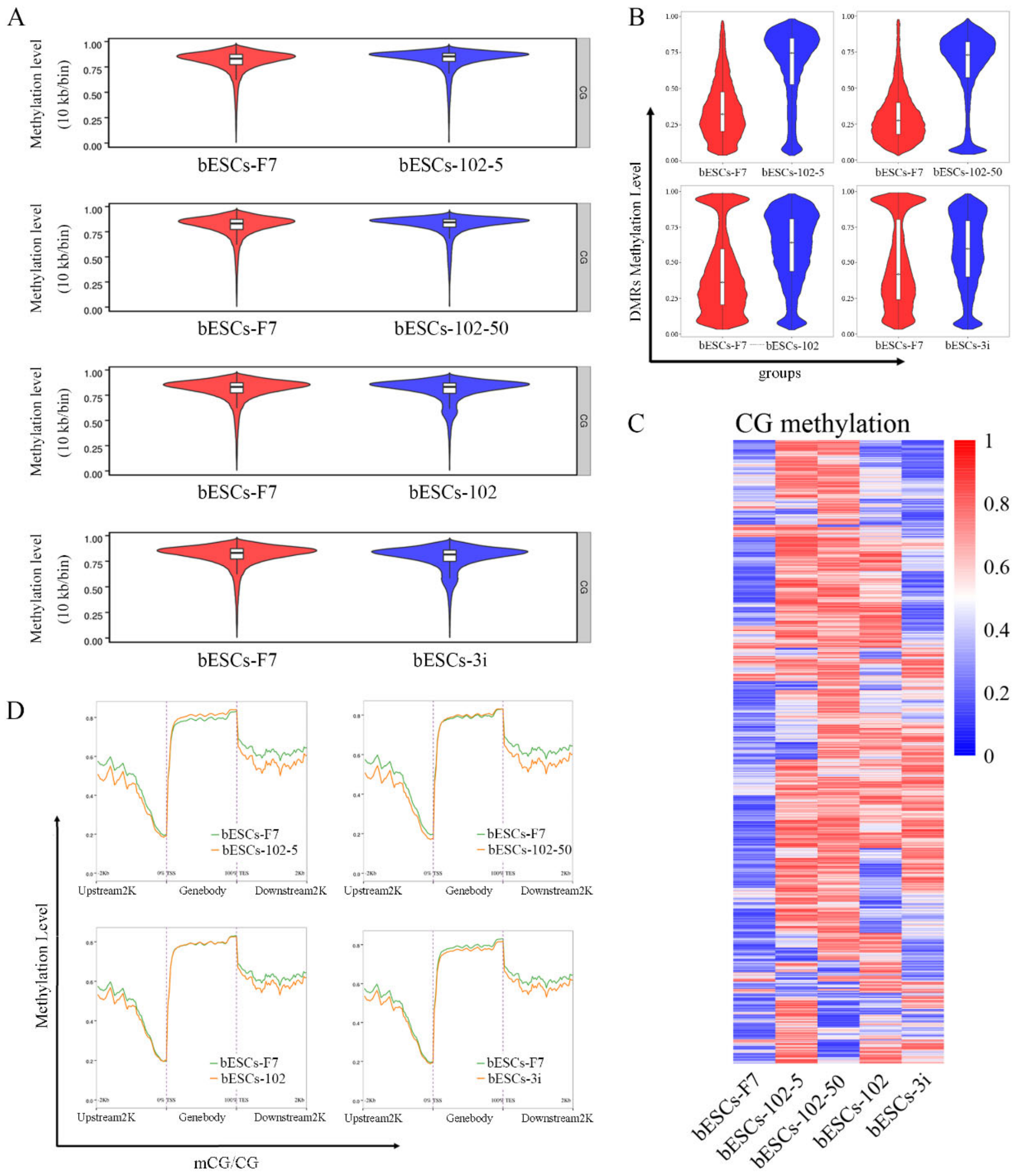

Figure S6. Differences of DNA methylation distribution of bESCs cell lines.

(A) Genome − wide horizontal distribution of methylation sites in of bESCs − F7, bESCs − 102 − 5 and bESCs − 102 − 50, bESCs − 102 and bESCs − 3i, with 10Kb as a bin, the width of each violin represents how much bin is at that methylation level. (B) CG DMR methylation horizontal distribution of bESCs − F7, bESCs − 102 − 5 and bESCs − 102 − 50, bESCs − 102 and bESCs − 3i. (C) Heat map of mCG of bESCs − F7, bESCs − 102 − 5 and bESCs − 102 − 50, bESCs − 102 and bESCs − 3i. DNA

methylation sequencing analysis was performed. RPKM values were used to define up − regulated genes (RPKM≥0.5, red) and down − regulated genes (RPKM <0.5, blue). (D) The methylation level of bESCs − F7, bESCs − 102 − 5 and bESCs − 102 − 50, bESCs − 102 and bESCs − 3i was 2K in the upstream and downstream of Genebody.

Table S1. Primers of qRT－PCR.

Genes	upstream primer	downstream primer
GAPDH	GGGTCATCATCTCTGCACCT	GGTCATAAGTCCCTCCACGA
OCT4	GGTTCTCTTTGGAAAGGTGTTC	ACACTCGGACCACGTCTTTC
SOX2	CATCCACAGCAAATGACAGC	TTTCTGCAAAGCTCCTACCG
NANOG	TTCCCTCCTCCATGGATCTG	ATTTGCTGGAGACTGAGGTA
NCAM1	AGAAGCAAGAGACCCTGGAC	AGAAGCAAGAGACCCTGGAC
TET1	AGAATGTCGGCTTGGGAAGA	TGGCTTCCATTCCTTCCCTT
TET3	GGAAGCGGTGTGGTACTTGT	GCTGAGCTCTGAGCCTGTCT
MEIS - 1	TGCAGGCAGTGTCTTAAGGA	CATGACCGATGCTTTGCTCA
FGF4	TACGGCTCGCCTTTCTTCAC	TTCTTGGCCTTGCCGTTCTT
GATA6	GCACCAGTATGGCTCGCT	CTCCAGCAGGTCTGTGCC
C - MYC	CCCATCAGCACAATTACGCA	TGTCCGCCTCTTGTCATTCT
KLF4	TCCCACCGCTCCATTAC	ATGAGAACTCTTCGTGTAGG
REX1	GGAAGAGGACCCACTCCTTC	ACTTGGCCTCCTAGTGCATC
TEAD4	ACTGGATCCAACAGGTGAGG	ATGTCAGAAGGGGTCAAACG
STELLA	TGCAAGTTGCCACTCAACTC	TTCCTTTGGCATAGCGAAGT
CD9	TTGGACTATGGCTCCGATTC	TGGCTGCAGCTACTTCAATG
BCL2	GATGACTTCTCTCGGCGCTA	GACCCCTCCGAACTCAAAGA
TFCP2L1	GTGCAGATCGACACCTTCAA	GGGAGCACTCTGAGAGGATG
HOXA9	CATCACCACCACCCCTATGT	GCGGTTCAGGTTTAATGCCA
MLL1	TGGAGCAGTCACCACAGAAG	TCACACCTGCAAATGAGAGC
PDRM14	CGGAGACAATTCCCTGATGT	CACGGGAATGTCCAGAAACT
DNMT3A	CTGGTGCTGAAGGACTTGGGC	CAGAAGAAGGGGCGGTCATC
DNMT3B	CCGCAGATCAAGCTCAC	GTTATTTCGGGTTCGGAC
DNMT3L	ATGAGCAACTGGGTCTGCTT	GGGCTCTCTCTTCCACACAG
DNMT1	AGTGGGGGACTGTGTTTCTG	TGTACGAGAGCTGCATGTCC

Table S2. Primer sets for BSP methylation assay

Genes	upstream primer	downstream primer
OCT4	GGTGTTGAGTAGTTTTTAGGAGAT TT	AAACCATCCCTCCACACAAATCA T
NANOG	GAAGGGATTGAAGGTTATTTGTT	ACACACCTTAAATAAACAAACC
